# Molecular evolution of the *pDo500 *satellite DNA family in *Dolichopoda *cave crickets (Rhaphidophoridae)

**DOI:** 10.1186/1471-2148-9-301

**Published:** 2009-12-28

**Authors:** Lene Martinsen, Federica Venanzetti, Arild Johnsen, Valerio Sbordoni, Lutz Bachmann

**Affiliations:** 1 National Centre of Biosystematics, Natural History Museum, University of Oslo, 0318 Oslo, Norway; 2 Via Giuseppe Berto 31, 00142 Rome, Italy; 3 Department of Biology, University of Rome Tor Vergata, Via della Ricerca Scientifica, 00133 Rome, Italy

## Abstract

**Background:**

Non-coding satellite DNA (satDNA) usually has a high turn-over rate frequently leading to species specific patterns. However, some satDNA families evolve more slowly and can be found in several related species. Here, we analyzed the mode of evolution of the *pDo500 *satDNA family of *Dolichopoda *cave crickets. In addition, we discuss the potential of slowly evolving satDNAs as phylogenetic markers.

**Results:**

We sequenced 199 genomic or PCR amplified satDNA repeats of the *pDo500 *family from 12 *Dolichopoda *species. For the 38 populations under study, 39 *pDo500 *consensus sequences were deduced. Phylogenetic analyses using Bayesian, Maximum Parsimony, and Maximum Likelihood approaches yielded largely congruent tree topologies. The vast majority of *pDo500 *sequences grouped according to species designation. Scatter plots and statistical tests revealed a significant correlation between genetic distances for satDNA and mitochondrial DNA. Sliding window analyses showed species specific patterns of variable and conserved regions. The evolutionary rate of the *pDo500 *satDNA was estimated to be 1.63-1.78% per lineage per million years.

**Conclusions:**

The *pDo500 *satDNA evolves gradually at a rate that is only slightly faster than previously published rates of insect mitochondrial COI sequences. The *pDo500 *phylogeny was basically congruent with the previously published mtDNA phylogenies. Accordingly, the slowly evolving *pDo500 *satDNA family is indeed informative as a phylogenetic marker.

## Background

Only a small fraction of a typical eukaryotic nuclear genome constitutes rDNA or protein-coding genes, whereas a large fraction of all higher eukaryotic genomes consists of repetitive sequences and heterochromatic satellite DNA (satDNA) [1,2]. Recent sequencing of various genomes have confirmed that the remarkable variability in genome size among eukaryotes is to a large extent due to different amounts of repetitive DNA, of which non-coding tandemly repeated DNA is a major part [3-7].

Typically, satDNA consists of numerous very similar repeated sequences, tandemly arranged in a head to tail orientation in large clusters up to 100 million bp in length [1]. These clusters are usually located in the heterochromatic parts of the chromosomes, mainly in the regions close to the centromeres and telomeres. Repeat size can vary largely within and between species; from only a few base pairs up to several thousand base pairs [8-11]. The biological significance of satDNA is still under discussion. No general function has been conclusively assigned to this genome component, although a number of possible functions have been put forward (summarized in [12]) out of which many challenge earlier ideas of satDNA being "junk" [13] or selfish "parasites" in the genome [14]. Most of the proposed functions of satDNA are related to heterochromatin and/or centromere formation and function. However, the enormous diversity of satDNA in nucleotide sequence, repeat length, complexity, and genomic abundance may indicate that satDNA indeed may have several different functions.

The evolutionary turnover of satDNA is usually very fast; i.e. in closely related species non-orthologous satDNAs are often found at homologous chromosomal locations [15]. However, other satDNA families evolve more slowly and are represented in several closely related species [16-18]. Some satDNAs even seem to be rather ancient and can be widely distributed among higher taxa [19,20]. Consequently, some satDNAs may be valuable taxonomic identification tools while others might be informative in phylogeny.

Most commonly, mitochondrial genes such as e.g., the ribosomal 12S and 16S, or the cytochrome oxidase subunit I (COI) and II (COII) genes [21,22], and/or nuclear ribosomal DNA (rDNA) sequences, such as the 18S and 28S genes, and the internally transcribed spacer (ITS) regions are applied as molecular markers. Nuclear protein-coding genes such as e.g., the elongation factor-1α (EF-1α), carbamyl-P synthetase/aspartate transcarbamylase/dihydroorotase (CAD), and wingless genes [23] have also been applied. Some molecular features such as e.g., an A/T bias at the third codon position of mitochondrial protein coding genes [24,25], and higher values of among-site rate variation [26] may limit the phylogenetic utility of standard markers under certain circumstances. Some studies have explored the potential of satDNAs as phylogenetic markers [18,27-33], but there is still no general agreement about the utility of satDNA in this context.

Here, we explored the mode of evolution of the specific *pDo500 *satDNA family in the cave cricket genus *Dolichopoda *Bolivar 1880 (Dolichopodinae, Rhaphidophoridae). Approximately 30 *Dolichopoda *species are patchily distributed throughout the North Mediterranean regions. They have colonized caves and hypogean habitats between the Pyrenees and the Caucasus. As most *Dolichopoda *species depend on natural caves, a high degree of geographical isolation and strictly allopatric speciation processes can be assumed [34]. This might allow insights into the short-term processes of genetic differentiation of *Dolichopoda *populations and species, and therefore makes the genus a suitable model for studying processes of molecular evolution of satDNA. Three specific satDNA families have previously been characterized for three geographically isolated populations of *D. schiavazzii *[35,36]. Two of them, the *pDoP102 *and *pDsPv400 *satDNA families, are species-specific whereas the *pDo500 *satDNA family occurs in the genomes of all *Dolichopoda *species studied so far. The *pDo500 *satDNA monomers have been found to include repeat motifs that resemble the structure of hammerhead ribozymes. *PDo500 *sequences are also transcribed to some extent and may perform self-cleavage. It has therefore been speculated that this satDNA may be under selective pressure [37].

The mitochondrial DNA phylogeny of *Dolichopoda *has been addressed in two recent studies [38,39]. Data on other markers, such as allozyme variability [40,41], single copy DNA-DNA hybridization [41], and RFLPs of mitochondrial DNA [42] are also available for many *Dolichopoda *species. These studies offer a solid background for interpreting the mode of evolution of satDNA in this genus. Here, we studied the *pDo500 *satDNA from twelve species of *Dolichopoda *in detail. We were also interested to assess whether or not a phylogenetic signal can be discerned from this satDNA family.

## Results

### Sequence composition and alignment

The nucleotide sequences of the *pDo500 *satDNA family in 12 *Dolichopoda *species were determined. We obtained 199 satDNA sequences with 3-9 sequences per population (Table 1). The length of the *pDo500 *sequences ranged from 463 bp to 505 bp. The total alignment consisted of 497 positions/characters (primer sequences excluded). There were 385 (77%) variable positions, 269 (54%) of which were parsimony informative. The average nucleotide composition was T = 35.3%, C = 24.6%, A = 21.7% and G = 18.4%. The transition/transversion rate ratios were *k*_1 _= 1.995 (purines) and *k*_2 _= 1.578 (pyrimidines). The overall transition/transversion bias was *R *= 0.87. The alignment of deduced population-specific consensus sequences consisted of 39 sequences and 483 positions/characters. Only for the PIL population of *D. geniculata *two specific consensus sequences were deduced due to too many ambiguous sites. There were 117 (24%) variable positions of which 52 (11%) were parsimony informative for the consensus sequence alignment. The average nucleotide composition of the deduced consensus sequences was almost identical to that of the total data set, i.e. T = 35.2%, C = 24.7%, A = 21.7% and G = 18.4%.

**Table 1:** *Dolichopoda *species and populations included in this study.

Species	**Pop**.	Locality	*pDO500 *sequences	Mean p-distance within populations	Mean p-distances between populations
*D. schiavazzii*	VET	Necropoli di Vetulonia, Grosseto, Toscana, Italy	5	0.024	0.019-0.059
	ORS	Grotta dei Pipistrelli, Montorsaio, Grosseto, Toscana, Italy	3	0.028	
	CPS	Monastero dei Fratelli Passionisti, Orbetello, Grosseto, Toscana, Italy	6	0.037	
	CIS	Acquedotto di Cisternino, Livorno, Toscana, Italy	6	0.056	
	BDO	Grotta di Buca dell'oro, Isola d'Elba, Grosseto, Toscana, Italy	7	0.015	
	POP	Populonia, Grosseto, Toscana, Italy	6	0.019	
	MRC	Marciana, Isola d'Elba, Grosseto, Toscana, Italy	6	0.030	
	FIC	Caverna di Fichino, Cascianna Terme, Pistoia, Toscana, Italy	9	0.065	
	BSC	Buca sopra cimitero, Orbetello, Grosseto, Toscana, Italy	5	0.016	
*D. aegilion*	CAM	Miniera di Campese, Isola del Giglio, Grosseto, Toscana, Italy	9	0.059	
*D. linderi*	SIR	Sirach Cave, Eastern Pyrenees, France	3	0.058	0.046-0.075
	MTB	Grotte de Montbolo, Montbolo, Eastern Pyrenees, France	4	0.031	
	BNP	Grotte de Bon Repaux, Bon Repaux, Ariege, Pyrenees, France	5	0.061	
	VMY	Grotte de Valmanya, Vinca, Eastern Pyrenees, France	6	0.055	
	CRQ	Mas de Crouanques, Pyrenees, France	8	0.030	
*D. bolivari*	FRN	Forat negre cueva, Serradel, Llerida, Pyrenees, Spain	5	0.030	
*D. cyrnensis*	VAT	Grotta di Valletto, Corsica, France	3	0.088	
*D. bormansi*	BRA	Grotta di Brando, Bastia, Corsica, France	3	0.075	
*D. baccetti*	PST	Grotta di Punta degli Stretti, Orbetello, Grosseto, Toscana, Italy	6	0.017	
*D. laetitia*	FOR	Ruderi di Villa Chigi, Formello, Roma, Lazio, Italy	4	0.015	0.015-0.019
	PSC	Grotta della Poscola, Monte di Malo, Priabona, Vicenza, Veneto, Italy	4	0.017	
	DIA	Grotta del diavolo, Semproniano, Grosseto, Toscana, Italy	4	0.011	
*D. palpata*	TRE	Grotta di Tremusa, Scilla, Reggio di Calabria, Calabria, Italy	4	0.072	
*D. capreensis*	CPR	Grotta San Michele, Isola di Capri, Napoli, Campania, Italy	5	0.056	
*D. geniculata*	PIL	Grotta la Pila, Poggio Moiano, Rieti, Lazio, Italy	6	0.039	0.030-0.058
	PAS	Grotta di Pastena, Pastena, Frosinone, Lazio, Italy	5	0.027	
	CLP	Grotta Regina Margherita, Collepardo, Frosinone, Lazio, Italy	6	0.041	
	TUS	Cunicolo dell'acquedotto, Frascati, Roma, Lazio, Italy	4	0.038	
	ISC	Fontana cunicoli, Isola di Ischia, Napoli, Campania, Italy	8	0.030	
	PRA	Grotta delle Praie, Lettomanoppello, Perugia, Umbria, Italy	5	0.044	
	VAL	Grotta Valmarino, Monte S. Biagio, Latina, Lazio, Italy	4	0.031	
	AUS	Grotta degli ausi, Prossedi, Latina, Lazio, Italy	5	0.030	
	PNZ	Le Forme, Isola di Ponza, Latina, Lazio, Italy	6	0.043	
	ZAN	Isola di Zannone, Latina, Lazio, Italy	5	0.027	
*D. ligustica*	COR	Buco del Corno, Valle Cavallina, Zandobbio, Bergamo, Lombardia, Italy	5	0.030	0.020-0.032
	PUG	Grotta del Pugnetto, Val di Lanzo, Torino, Piemonte, Italy	6	0.015	
	SFL	Grotta Selva, Zandobbio, Bergamo, Lombardia, Italy	4	0.026	
	BOS	Grotta di Bossea, Frabosa Soprana, Cuneo, Piemonte, Italy	5	0.029	

The number of *pDo500 *sat DNA sequences per population and the mean genetic distance within and between populations (uncorrected p-distances) are listed.

### Sliding window analyses

**Figure 1:**
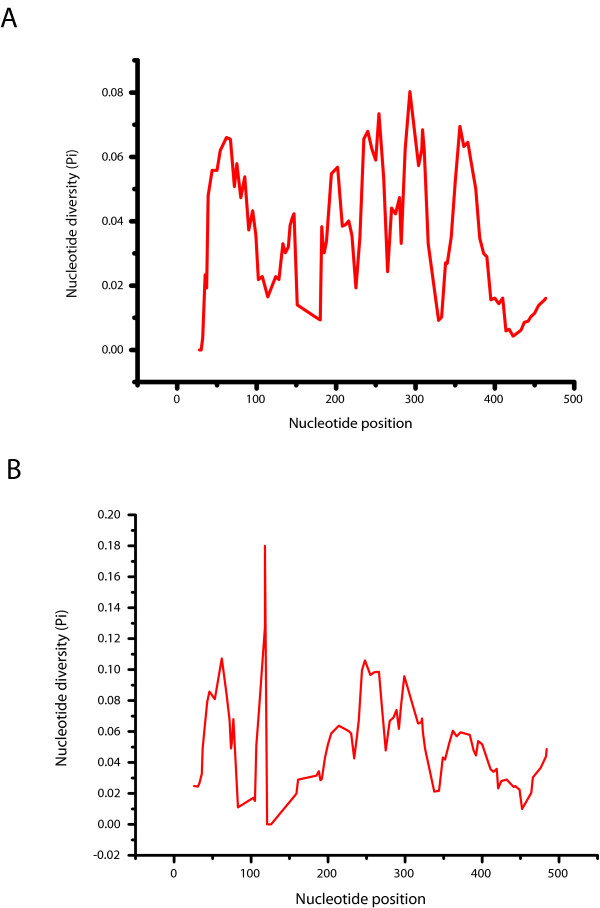
**Sliding window analyses of the *pDo500 *satellite DNA of all *Dolichopoda *species included in this study**. The results are shown for both the consensus alignment (A) and the complete alignment (B). The graphs show the value of nucleotide diversity (π) in a sliding window size of 30 with step size 5. Each value is depicted at its mid-point.

In order to identify regions within the *pDo500 *satDNA family with high levels of intra- and interspecific sequence conservation, sliding window analyses were performed. As shown in Figure 1A, nucleotide diversity (π) ranged between 0.009 and 0.093 for the consensus alignment. Under the applied settings, there were three regions with relatively low local minima of π (0.017, 0.009, and 0.014) at window midpoint positions 180, 329, and 424, respectively. Conversely, there were four peak areas with relatively high π values (0.075, 0.076, 0.080 and 0.093) with local maxima at window midpoint positions 202, 292, 357 and 239, respectively. When analyzing the complete alignment that also takes into account the intrapopulational variation, there were three regions with relatively low local minima of π (0.011, 0.00, and 0.010) at window midpoint positions 83, 121, and 452, respectively (Figure 1B). The peaks indicating regions with high local maxima were detected at positions 62 (π = 0.107), 118 (π = 0.180), 248 (π = 0.106), and 299 (π = 0.096). At first glance the extremely low π value around window midpoint position 121 in the total alignment may be surprising because this low local variation is not reflected in the consensus alignment to that extreme. However, the "discrepancy" can be assigned to different arrangements of indels in the two alignments and some ambiguous sites in the consensus sequences. It is also worthwhile mentioning that the area in the sliding window analysis that corresponds to the region that can form a hammerhead-like structure [37] does not show higher levels of conservation than the rest of the sequence.

**Figure 2:**
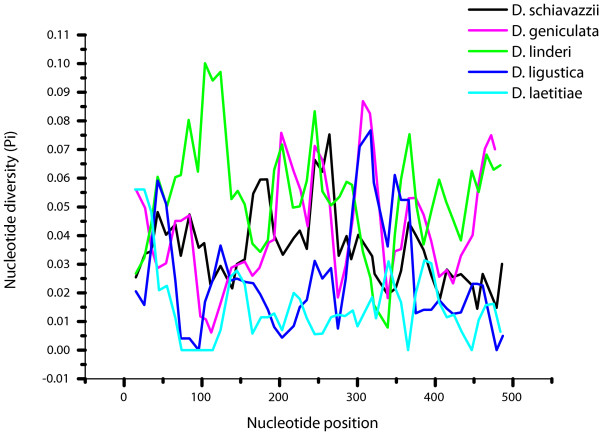
**Sliding window analyses of the *pDo500 *satellite DNA family of five *Dolichopoda *species separately**. The graphs show the value of the nucleotide diversity (π) in a sliding window size 30 with step size 10. Each value is depicted at its mid-point.

The sliding window analyses for selected species that are represented by a reasonable number of populations and individual *pDo500 *sequences are shown in Figure 2. Five areas with high interspecific variability were identified: i) For positions 66-124 *D. laetitiae *was significantly different from the other species (p < 0.05); ii) For positions 200-250 *D. linderi *and *D. geniculata *differed significantly from the other species (p < 0.05) but not from each other; iii) For positions 300-320 *D. linderi *and *D. laetitiae *were significantly different from the other species and from each other (p < 0.05); iv) For positions 350-380 *D. linderi *and *D. laetitiae *are significantly different from the other species and from each other (p < 0.05); and v) For positions 447-490 *D. geniculata *and *D. linderi *are significantly different from the other species (p < 0.05) but not from each other.

### Genetic distance analyses

The mean K2P and uncorrected p distances of the *pDo500 *satDNA sequences within and between populations as well as between species are given in Additional files 1 and 2, respectively.

**Figure 3:**
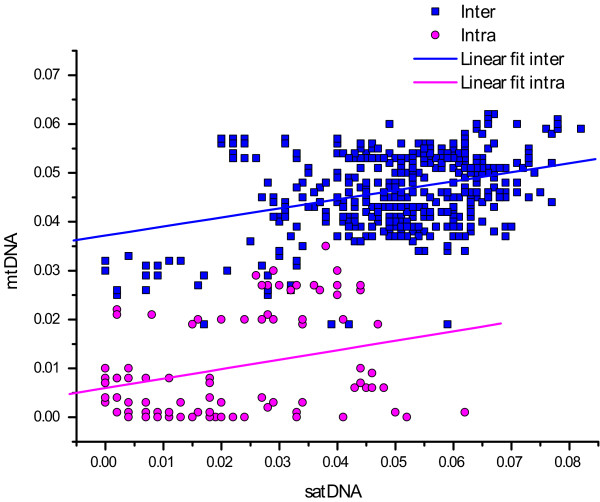
**Scatter plot of inter- and intraspecific genetic distances (uncorrected p-distances), blue squares and red circles, respectively**. Trend lines from linear regression analyses are shown for visual purposes. The linear regression lines are Y = 0,005977 + 0.193514X and Y = 0,037182 + 0.184845X for the intra- and interpopulational genetic distances, respectively.

The scatter plot (Figure 3) relates the mean interpopulational uncorrected p-distances of *pDo500 *satDNA sequences to the average mtDNA distances calculated from partial 16S and COI sequences [38,39]. There was a highly significant correlation between genetic distances based on the two molecular markers (Mantel test: r = 0.616, p = 0.0001). The Spearman Ranks Correlation tests revealed that also the interpopulational genetic distances within and between species of the *pDo500 *satDNA and the mtDNA were correlated, Rs = 0.234 (p < 0.0001) and Rs = 0.231 (p = 0.03), respectively. The biological relevance of the correlations from the Spearmans Ranks test might be debated due to the low Rs values. There was also a significant correlation between the genetic distances based on satDNA and the geographic distances between the populations (Mantel test: r = 0.274, p = 0.0001). Despite the observed significant overall association, it is noteworthy that for the *pDo500 *satDNA, a number of intraspecific genetic distance values are higher than some of the interspecific values, e.g. between some *D. linderi *populations and some *D. schiavazzii *populations. On the other hand, some interspecific genetic distance values are quite low, e.g. *D. laetitiae *(DIA) vs *D. geniculata*. For comparison, for the mitochondrial DNA the vast majority of the intraspecific genetic distance values are lower than the interspecific values.

The saturation plot with uncorrected P-distances versus GTR-distances yielded a literally straight line (data not shown). Separate plots with the transition and transversion ratios showed two straight lines, transversions being more frequent than transitions (data not shown). This plot indicated that there was no substitution saturation in the *pDo500 *data set.

### Phylogenetic analyses

**Figure 4:**
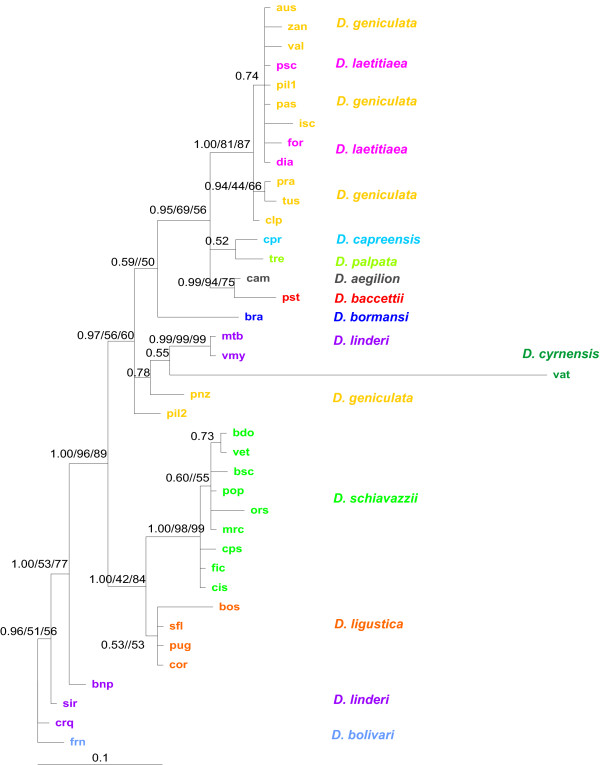
**Bayesian phylogeny of the 39 derived consensus sequences the *pDo500 *satellite DNA family of *Dolichopoda *cave crickets**. The Parsimony and Maximum Likelihood analyses yielded largely congruent trees. Posterior probabilities (PP) and bootstrap support > 50 from the MP and ML analyses are given for each node in the following order PP/MP/ML. Abbreviations for populations are as in Table 1.

The phylogenetic hypothesis depicted in Figure 4 resulted from the Bayesian analysis of the consensus alignment of the *pDo500 *satDNA sequences. The respective tree for the entire dataset is provided in Additional file 3. In order to allow for a comparison with the previously published mitochondrial DNA phylogenies [38,39], the satDNA tree was rooted with *D. bolivari*. The three different phylogenetic analyses (MP, MrBayes and ML) resulted in very similar tree topologies with only a few minor incongruencies. The MP analysis yielded 425 trees of equal length (252 steps) whereas ML analysis resulted in two trees; in both cases, the 50% majority-rule consensus trees were congruent with the phylogenetic hypothesis obtained with the Bayesian analysis.

As shown in Figure 4, the *pDo500 *sequences clustered in three main groups in the Bayesian tree: 1) *D. bolivari *and the majority of the sequences from *D. linderi*, 2) *D. ligustica *and *D. schiavazzii*, and 3) *D. geniculata, D. laetitiae*, *D. aegilion, D. baccettii, D. cyrnensis, D. capreensis *and *D. palpata*, in addition to two sequences from *D. linderi *(VMY and MTB). In contrast to high posterior probability values in the Bayesian analysis the bootstrap support values from the MP analysis are quite low for many nodes.

The phylogeny obtained with the complete *pDo500 *alignment (Additional file 3) is congruent with the phylogeny of the consensus alignment. However, there are also some sequences that do not cluster conspecifically, which reflected the intraspecific variation among *pDo500 *repeats. To some extent, sequences from the same population cluster together, but there is little intraspecific structure in the phylogeny of the complete alignment. This means that hardly any population-specific signatures could be attributed to the *pDo500 *sequences.

### Evolutionary rate estimates

The *pDo500 *sequences evolve on average 1.48 times faster than the COI sequences from *Dolichopoda*. Multiplied by the previously reported substitution rate for insect COI genes of 1.1-1.2% per lineage per million years, per silent sites [43], this gives an evolutionary rate of 1.63-1.78% per lineage per million years for the *pDo500 *sequences.

The congruency of the satDNA dataset and the previously published mtDNA dataset were evaluated by a partition homogeneity test/ILD and Partitioned Bremer Support (PBS) values. Three different tests were performed for the ILD: i) *pDo500 *+ COI, ii) *pDo500 *+ 16S, and iii) *pDo500 *+ COI + 16S. The null hypothesis of homogeneity of the phylogenetic signal among the data sets were rejected for all three tests (p = 0.001). This may indicate that the two data sets reflect different phylogenetic signals that should yield incongruent phylogenetic trees. PBS values were determined for one of the MPTs, the strict consensus and the 50% majority rule consensus tree. The strict consensus tree did not yield informative PBS values probably due to lack of structure in the tree, i.e. too many polytomies. The PBS values for the MPT and the 50% MJR consensus tree (Additional files 4 and 5) were to some extent indicating conflict between the two datasets and thus in line with the results of the ILD tests. However, the satellite DNA and the mtDNA sequences do not contribute equally to the tree. The mtDNA sequences are approximately three times as long as the *pDo500 *satDNA sequences and accordingly contribute more parsimony informative sites (296) than the *pDo500 *satDNA consensus sequences (52). Nodes with PBS values indicating strongest conflict were usually those with both low bootstrap support and posterior probabilities in the phylogenetic analyses. Not surprinsingly, for nodes with strong statistical support in the phylogenetic analyses, the PBS values indicated only little conflict. However, conflicting PBS values are certainly to some extent due to the high number of equally parsimonious trees obtained in the analysis of the combined data set as described by Lambkin et al. [44].

## Discussion

In the current paper, we study the mode of evolution of the tandemly arranged satellite DNA family *pDo500 *in *Dolichopoda *cave crickets. Scatter plots and the related statistical analyses showed a significant correlation between the K2P distances calculated for mtDNA and the *pDo500 *satDNA. The *pDo500 *sequences evolve on average 1.48 times faster than the COI sequences from *Dolichopoda*. This is lower than previously observed for Hawaiian *Tetragnatha *spiders, for which a four times increased nucleotide substitution rate has been reported for satDNA compared to mtDNA [30]. The among-site rate variation of the *pDo500 *was higher than estimated for mtDNA from the same species, which may indicate that some regions of the *pDo500 *sequences are under selection. Accordingly, previous observations on preliminary data from populations of the *D. laetitae-geniculata *complex already indicated that this family of sequences is not informative at the intraspecific level [45]. As has been reported for most satDNA families the *pDo500 *family is slightly AT-rich (~57%), which is also common for mtDNA. Nevertheless, homoplasy is expected to be limited for the *pDo500 *satDNA family because no saturation was detected. Whether or not the *pDo500 *family is a typically slowly evolving satDNA with respect to among-site rate variation and saturation is difficult to assess. Among-site rate variation has hardly ever been reported for satDNA families, and saturation tests have only rarely been performed, e.g. in Pons et al. (2002) [29] who report slight saturation for the *PIM357 *satDNA family in *Pimelia*. Nevertheless, the high among-site rate variation did not obscure the phylogenetic signal in the *pDo500 *satDNA sequences. This conclusion may to some extent contradict the results of the sliding window analyses. For at least some species such as e.g. *D. ligustica, D. schiavazzii*, and *D. linderi *the sliding window profiles differed significantly.

### *pDo500 *satDNA based phylogeny in *Dolichopoda*

The phylogeny obtained with the *pDo500 *satDNA (Figure 4 and Additional file 3) is largely congruent to those previously published on the basis of mtDNA markers [38,39]. The main differences between these phylogenies can be summarized as follows: i) Two well supported clusters in the mtDNA tree appear in different positions in the satDNA phylogeny. These clusters are *D. geniculata *+ *D. laetitiae *and *D. schiavazzii*, which swap positions. ii) With satDNA as marker the two Corsican species *D. cyrnensis *and *D. bormansi *do not cluster together. iii) In two cases (*D. linderi *and *D. geniculata*) satDNA consensus sequences representing different populations do not cluster conspecifically.

In earlier studies on the phylogeny of *Dolichopoda *using the mitochondrial 16S and cytochrome oxidase I genes, the northern Italian *D. ligustica *grouped with the Central- and Southern-Italian *D.laetitiae-geniculata *complex. With respect to biogeography this grouping was difficult to explain [39]. However, in the satDNA phylogeny, *D. ligustica *grouped with the more northern species *D. schiavazzii*. Similarly, the two southernmost species *D. palpata *and *D. capreensis *grouped with the Northern Italian *D. schiavazzii *in the mtDNA phylogeny, which apparently did not conform to the biogeographical pattern. In contrast, the satDNA phylogeny indicates that *D. palpata *and *D. capreensis *are closely related to *D. laetitae-geniculata *complex that is also geographically closest. Thus, we believe that at least in these instances, the tree topology obtained with the *pDo500 *data is a better reflection of the *Dolichopoda *phylogeny than those obtained with mtDNA markers.

However, *D. baccettii *and its sister taxon *D. aegilion *are geographically more close to *D. schiavazzii*, but in the satDNA phylogeny, as opposed to the mtDNA phylogeny, *D. baccettii *and *D. aegilion *are found closely related to *D. laetitiae-geniculata*. This is in line with the results of a preliminary study [46]. Interestingly, the *D. laetitiae-geniculata *complex is also found in the mid-Italian region as is *D. baccettii*, therefore, the relationships found in the satDNA tree may be plausible. However, morphological features are more in favour of the phylogeny as resolved by mtDNA sequences, since *D. schiavazzii*, *D. bormansi *and *D. cyrnensis *share spinulation on the femurs and are grouped in the same subgenus *Chopardina*. Furthermore, *D. bormansi*, and *D. cyrnensis *have been recorded from the same caves and even hybridization of the two species has been hypothesized [47]. The incongruence between the satDNA and mtDNA may have been expected at least according to the results of the sliding window analyses. These results indicated that the intra- and interspecific variation of different regions of the *pDo500 *repeats differ significantly. Accordingly, among-site rate variation in the dataset was high. However, the incongruencies may also be due to stochastic processes in the usually small *Dolichopoda *populations such as random sorting of ancestral lineages during the short internodes, homoplasy in the mtDNA data, or both. The processes of molecular drive (see [48] for a review on molecular drive) affecting the *pDo500 *satDNA may also lead to an accumulation of variants that do not perfectly reflect the phylogenetic relationships.

As mentioned above, the level of the among-site rate variation of the *pDo500 *is higher (α = 0.59) than estimated for mtDNA (α = 0.88). This could be due to selection pressure on the region where the previously reported hammerhead structure is found [37]. However, according to the sliding window analyses performed here, this region is overall not more conserved than the rest of the *pDo500 *repeat. Nevertheless, since it is the secondary structure that is most crucial to the hammerhead and not the sequence itself, we can not rule out that the potential hammerhead structure has a profound influence on the evolution of the *pDo500 *satDNA family.

The congruence of the phylogenies obtained with the *pDo500 *satDNA and mtDNA markers is to some extent contradicted by the results of the ILD test and the PBS values. The partition homogeneity test indicates disagreement between the phylogenetic signal in the data sets based on mtDNA and satDNA. However, several authors have questioned the validity of this test as a criterion for congruence and combinability [49,50]. It has been demonstrated that the ILD test can wrongly reject the null hypothesis of congruence if the two data sets compared have different among-site rate variation [51]. Whether this applies to our data sets is uncertain as the satDNA and the mtDNA data sets differ only slightly in among-site variation. However, the ILD test is also sensitive for homoplasy, meaning random noise that is unequally distributed between the partitions [50]. There is homoplasy in both data sets due to little intraspecific structure; for example, within *D. schiavazzii *the populations can change position in the tree topology without altering the tree length. In our dataset a number of species were represented by several populations all with very similar if not identical *pDo500 *satDNA sequences. It is thus not surprising that parsimony analyses yielded many equally parsimonious trees. However, this may lead for several nodes to PBS values indicating conflicts between the combined satDNA and mtDNA datasets. However, it should be noted that nodes with PBS values indicating strongest conflict were usually those with both low bootstrap support and low posterior probabilities in the phylogenetic analyses.

Our study has added to the evidence that there is a clear potential for using slowly evolving satDNA families as phylogenetic markers. This is in line with a number of previous studies e.g. [17,18,30,52] that also used consensus sequences of satDNA and obtained phylogenies that were largely congruent with those based on other markers. In addition, several studies have successfully used satDNA as taxonomic and phylogenetic markers to solve issues that other markers such as e.g., mtDNA markers could not resolve [53,54].

There is an extensive literature on the characteristics of molecular phylogenetic markers e.g. [22,26,55-65], and at least at first glance satDNA seems to violate all requirements. In brief, a phylogenetic molecular marker must show an appropriate level of sequence conservation for the taxa of interest, while at the same time providing a sufficient number of variable and informative sites. Ideally, all sites should vary with equal probability because high among-site rate variation might hamper obtaining the true phylogeny. Furthermore, equal base composition will keep homoplasy low. Single-copy sequences are preferred in order to avoid paralogous comparisons. Finally, methodological aspects, such as ease to amplify by PCR or the availability of universal primers, are relevant as well. No marker will satisfy all of the above criteria and systematists therefore have developed a toolbox of most commonly used markers.

In comparison to protein-coding and ribosomal genes, the evolutionary turnover of non-coding satDNA is usually very high, leading to marked sequence divergence between species. Thus, there is often little or no phylogenetic signal when comparing satDNA from closely related species e.g. [66]. Accordingly, two species-specific satDNAs have earlier been described also for *Dolichopoda schiavazzii *[16,36]. For the *pDo500 *satDNA family, we have also shown high among-site rate variation, and for tandemly repeated satDNA paralogous comparisons are certainly an issue. In addition, there are no universal satDNAs and slowly evolving satDNA families to be used as phylogenetic markers need to be identified case by case. Nevertheless, several satDNAs such as the *pDo500 *satDNA family have been described as gradually evolving, and are conserved over considerable evolutionary time. Such satDNA may be phylogenetically informative [16,17,29,67,68]. For the *pDo500 *satDNA family, once sequences were available, the alignment of repeats was straightforward because there were only few indels.

## Conclusion

Most satellite DNAs described so far have high evolutionary turn-over rates leading to rapid changes between species (interspecific heterogeneity) in contrast to homogenization and fixation within species (intraspecific homogeneity). However, as discussed here, some satDNA families such as the *pDo500 *satDNA family of *Dolichopoda *cave crickets may evolve rather slowly with an evolutionary rate only slightly faster than mtDNA. In *Dolichopoda*, both the fast evolving and the more slowly evolving satDNA have been found. In *D. schiavazzii*, two species-specific satDNA families have been identified, the *pDoP102 *(102 bp) and *pDsPv400 *(400 bp) in addition to the genus-specific family of *pDo500 *studied here [35,36]. These three satellite DNA families differ significantly in their molecular characteristics. A comparison showed a trend of sequence variability and copy number being positively correlated, and a trend of sequence variability and length of repeat being negative correlation [36], but data sets from further species are needed in order assess if these trends reflect general patterns. Unfortunately, very few other examples are found in the literature with both fast-evolving and slowly-evolving satDNAs found within the same species e.g. [69,70].

For some satDNA families a high degree of conservation was observed for species that diverged millions of years ago [52,71-73]. Such slowly evolving satDNA can even be a useful tool for phylogenetic analyses at higher taxonomic levels. Our study illustrates that satellite DNA can be successfully used as a molecular marker in phylogenetic analyses. In a phylogeographic context we believe that the *pDo500 *in some cases yields even better hypotheses than mtDNA. Compared with other commonly used markers - mtDNA and nuclear protein-coding and rDNA genes - particular gradually evolving satellite DNA families may fulfil the criteria of a good phylogenetic marker satisfactorily.

## Methods

### Material

We included samples from 38 populations representing 12 *Dolichopoda *cave cricket species from Italy and Spain (Table 1). The species' distributions are given in Additional file 6. Previously reported sequences of the *pDo500 *satDNA family from three populations of *D. schiavazzii *(VET, CPS, and BDO) [36] were also included in the analyses.

### DNA extraction, PCR, cloning and sequencing

Genomic DNA was extracted from femurs and heads of *Dolichopoda *using the QIAamp DNA purification kit (Qiagen) following the manufacturer's instructions, or by standard procedures as described in Sambrook et al. [74].

Sequences of the *pDo500 *satDNA family used in this study were obtained by two different approaches: i) Genomic DNA was digested with the restriction enzyme *Pst*I and subsequently electrophoresed on 5% polyacrylamide gels. The ladder-like strongly stained bands were cloned into standard plasmid vectors such as e.g., pUC19 [74]. Plasmid preparation was done according to the protocol of the QIAprep Spin Miniprep Kit (Qiagen). This approach can only discover *pDo500 *copies containing the *Pst*I cleavage site. ii) Copies of the *pDo500 *satDNA family were amplified from genomic template DNA using the primers 5'-GTTTTACACGTTCACTGCAG-3' and 5'-GACACATTGATGAGACTGCAG-3' [36]. The obtained PCR products were cloned using the Zero Blunt^® ^TOPO^® ^Cloning Kit (Invitrogen). Positive clones were selected through PCR amplification using the M13 forward and M13 reverse primers. Sequencing was performed on an ABI 3100 automatic sequencer using BigDye chemistry (Applied Biosystems). This approach may be biased by preferential annealing of the primers to certain *pDo500 *variants.

All sequences have been deposited in GenBank, accession numbers: GU322143-GU322341

### Sequence Analyses

The sequences were aligned using the software BioEdit [75]. Due to the high level of sequence similarity aligning all sequences was straightforward. The primer sequences of the PCR derived *pDo500 *repeats were excluded from the subsequent analyses.

Following the concept of concerted evolution [76], population-specific consensus sequences were deduced manually by evaluating each position in the alignment according to the six classes of transitional stages in tandem repetitive DNA described by Strachan et al. [77]. The different stages represent various steps in the homogenization and fixation process amongst repeats of tandemly repeated DNA families between pairs of species and populations. In essence this means that we used a 50% majority rule to deduce the consensus sequence. In some cases we either used the standard IUPAC symbols for ambiguous sites or deduced more than one consensus sequence for the respective populations.

Nucleotide composition, number of variable and parsimony informative sites, the transition/transversion bias, and genetic distance values were calculated using Mega version 4.0.1 [78]. All positions containing alignment gaps and missing data were excluded in pair-wise sequence comparisons.

### Phylogenetic reconstruction and statistics

In order to assess whether nucleotide substitutions reach saturation, both transitions and transversions, and uncorrected genetic distances (p-distance), were plotted in Microsoft Excel against distances based on the general-times reversible model (GTR). The GTR distances were calculated in PAUP through the Bioportal at the University of Oslo, Norway http://www.bioportal.uio.no/.

Phylogenetic analyses were conducted using Bayesian, Maximum Likelihood (ML), and Maximum Parsimony (MP) approaches. The phylogenetic analyses were performed on both the complete alignment and the consensus sequences. The appropriate substitution models were determined using MrModeltest [79]. For both alignments, the general time reversible substitution model with gamma distribution for the among-site rate variation (GTR+Γ) obtained highest score according to the Akaike information criterion. The shape parameter of the gamma distribution (α) is inversely related to the rate of variation, and low values (α < 0.5) suggest extreme rate heterogeneity [80]. Accordingly, for the *pDo500 *sequences α = 0.59 suggests a relatively high rate heterogeneity.

The Bayesian phylogenetic analysis was conducted using MrBayes [81]. Each analysis was run with 6 million generations, 4 chains (one cold, three heated) and a sampling frequency of 100. A 50% majority rule consensus tree was made from each analysis with the first 12,000 trees ignored as burn-in. The ML using PAUP [82] was conducted through the Bioportal at the University of Oslo, Norway http://www.bioportal.uio.no/. MP was done using the program TNT (Tree analysis using New Technology) [83] made available online with the sponsorship of the Willi Hennig Society http://www.cladistics.org/tnt.html. The TNT searches were conducted with gaps being treated as fifth character state. Bootstrap support values [84] were estimated for both the ML analysis and the MP analyses, with 500 and 1000 repetitions respectively.

The evolutionary rate of the *pDo500 *satDNA family was estimated in relation to a previously reported substitution rate (1.1-1.2% per lineage, per million years, per silent sites) for insect mitochondrial COI sequences [43]. Interspecific K2P distances [85] for the *pDo500 *satDNA data set were related to interspecific genetic distances for the COI data set from the same species in order to estimate the relative evolutionary rate. The species specific average multiplied by the 1.1-1.2% rate estimate yielded the evolutionary rate of *pDo500*. These estimates include all *pDo500 *sequences obtained in this study.

Sliding window analyses were performed in DnaSP [86] in order to detect regions of high sequence conservation. In DnaSP, gaps are not treated as a fifth character state when analyzing the data, but in the sliding window analysis it is possible to consider sites with gaps in the length of the windows. We conducted the analyses with both options. The window sizes were set as 50, 30 and 20, respectively, with three different step sizes, 1, 5 and 10, for all three window sizes. The analysis was performed on both the consensus alignment and the complete alignment. Since the sliding window analysis in DnaSP can not handle more than 181 sequences, our complete alignment needed to be slightly reduced. Sequences to be excluded from the analysis were randomly chosen from those populations with the highest number of sequences. We also analyzed the sequences of *D. schiavazzii, D. geniculata, D. linderi*, and *D. ligustica *separately. Since DnaSP does not handle standard IUPAC symbols for ambiguous sites, such positions in the consensus alignment were replaced by N's.

From the sliding window analyses of *D. schiavazzii, D. geniculata, D. linderi*, *D. laetitiae *and *D. ligustica*, regions in the *pDo500 *satDNA sequences with high variability between the species were identified. Uncorrected p-distances within each species were calculated for these regions [78], and subjected to an Analysis of Variance (Kruskal-Wallis ANOVA) with subsequent Post-hoc tests - Tukey honest significant difference test for unequal sample size [87]. The tests were performed in Statistica [88].

The congruence of the satDNA and the mitochondrial DNA data sets was addressed with a partition homogeneity test/incongruence-length difference test (ILD) and by Partitioned Bremer Support (PBS). The ILD test was performed in PAUP [82] and TNT; the Partitioned Bremer Support was obtained using TreeRot [89] in combination with PAUP (following the instructions of the TreeRot manual). In PAUP, the number of ILD replicates was set to 1,000, with 10 random addition sequence replicates, holding one tree per replicate. A time limit for each replicate was set to 200 seconds. In TNT, the analysis was done with a script provided by Pablo Goloboff with some modifications [90]. The number of replicates was set to 10,000, with 10 random addition sequences holding 10 trees per replicate, and branch swapping with TBR (tree bisection-reconnection). For calculating the PBS parsimony analysis was conducted in PAUP on the satDNA consensus sequences combined with the mtDNA sequences. The analysis yielded 14,232 most parsimonious trees (MPTs). For the analysis in TreeRot, we used both of the consensus trees and one of the equally parsimonious MPTs (the first one to appear in the tree file from PAUP).

Scatter plots showing interpopulational and interspecific distances from *pDo500 *sequences versus mitochondrial DNA sequences were made in Microsoft Excel. Two different plots at each taxonomic level were made: one using all the *pDo500 *sequences, and one using only the consensus sequences. The results were similar and we only present the plot with the consensus sequences.

Two Mantel tests were performed with 10,000 random iterations using Mantel2 [91]: i) to test the correlation between genetic distances of satDNA and genetic distances of mitochondrial DNA; and ii) to test the correlation between genetic distances of satDNA and geographic distances between populations. P-distances between populations [78], were used as input data for the genetic distances. The program ArcView Gis 3.3 was used to obtain geographic distances.

Spearman Rank Correlation tests were performed in order to assess the correlation between genetic distances based on satDNA and mtDNA in within- and between species comparison, respectively. The tests were performed using the Free Statistics and Forecasting Software at http://www.wessa.net[92].

## Authors' contributions

LB, FV and VS planned the project, conducted labwork, and participated in writing and editing the manuscript. LM conducted the labwork, analyzed the data and participated in writing and editing the manuscript. AJ participated in data analyses and writing and editing of the manuscript. All authors approved the final version of the manuscript.

## Supplementary Material

Additional file 1 **Interspecific genetic distances of the *pDo500 *satDNA sequences**. Kimura (1980) two-parameter distances (above diagonal) and uncorrected p-distance (below diagonal) of 199 genomic or PCR amplified *pDo500 *satDNA sequences from *Dolichopoda*. Click here for file

Additional file 2 **Interspecific genetic distances based on *pDo500 *satDNA consensus sequences**. Interspecific Kimura (1980) two-parameter distances (above diagonal) and uncorrected p-distance (below diagonal) of 32 derived population specific *pDo500 *satDNA consensus sequences from *Dolichopoda* Click here for file

Additional file 3 **Unrooted Bayesian phylogeny of the 199 repeats of the *pDo500 *satDNA family of *Dolichopoda *cave crickets**. Posterior probabilities (PP) > 50% are given. Abbreviations for populations are as in Table 1. The Maximum Parsimony and the Maximum Likelihood analyses were congruent with the Bayesian analysis. Click here for file

Additional file 4 **Partition Bremer Support values for one of the most parsimonious trees from the *pDo500 *satDNA consensus and the mtDNA sequences**. The PBS values for the two data partitions are given as satDNA/mtDNA. Click here for file

Additional file 5 **Partition Bremer Support values for the 50% majority-rule consensus tree derived for the *pDo500 *satDNA consensus and the mtDNA sequences**. The 50% majority-rule consensus tree is based on the MPTs from the combined parsimony analysis of the satDNA consensus and the mtDNA sequences. The PBS values for the two data partitions are given as satDNA/mtDNA. Click here for file

Additional file 6 **Map of the geographical distributions of *Dolichopoda *species included in this study**. The map is showing the distribution areas for the *Dolichopoda *species in the West Mediterranean region. Click here for file
