# Supplementary material for: Molecular evolution of the pDo500 satellite DNA family in Dolichopoda cave crickets (Rhaphidophoridae)
Source: BMC Evol Biol. 2009 Dec 28;9:301. doi: 10.1186/1471-2148-9-301 (PMC2808323; doi:10.1186/1471-2148-9-301)
Supplement: Additional file 1 — Interspecific genetic distances of the pDo500 satDNA sequences. Kimura (1980) two-parameter distances (above diagonal) and uncorrected p-distance (below diagonal) of 199 genomic or PCR amplified pDo500 satDNA sequences from Dolichopoda. [file 1471-2148-9-301-S1.DOC]

**Additional file 1.** Interspecific genetic distances. Kimura (1980) two-parameter distances (above diagonal) and uncorrected p-distance (below diagonal) of 199 genomic or PCR amplified *pDo500* satDNA sequences from *Dolichopoda.*

|  | **Species** | ***1*** | ***2*** | ***3*** | ***4*** | ***5*** | ***6*** | ***7*** | ***8*** | ***9*** | ***10*** | ***11*** | ***12*** |
| --- | --- | --- | --- | --- | --- | --- | --- | --- | --- | --- | --- | --- | --- |
| **1** | ***D. schiavazzii*** |  | 0.093 | 0.076 | 0.070 | 0.121 | 0.096 | 0.077 | 0.079 | 0.088 | 0.089 | 0.078 | 0.049 |
| **2** | ***D. aegilion*** | 0.087 |  | 0.088 | 0.094 | 0.117 | 0.089 | 0.051 | 0.065 | 0.079 | 0.069 | 0.069 | 0.084 |
| **3** | ***D. linderi*** | 0.072 | 0.082 |  | 0.052 | 0.131 | 0.088 | 0.072 | 0.077 | 0.077 | 0.083 | 0.076 | 0.063 |
| **4** | ***D. bolivari*** | 0.066 | 0.088 | 0.050 |  | 0.137 | 0.086 | 0.078 | 0.074 | 0.077 | 0.086 | 0.074 | 0.056 |
| **5** | ***D. cyrnensis*** | 0.110 | 0.108 | 0.119 | 0.124 |  | 0.140 | 0.120 | 0.120 | 0.128 | 0.123 | 0.123 | 0.121 |
| **6** | ***D. bormansi*** | 0.089 | 0.083 | 0.083 | 0.081 | 0.127 |  | 0.078 | 0.079 | 0.079 | 0.084 | 0.079 | 0.079 |
| **7** | ***D. baccettii*** | 0.073 | 0.048 | 0.069 | 0.074 | 0.110 | 0.074 |  | 0.051 | 0.067 | 0.060 | 0.054 | 0.070 |
| **8** | ***D. laetitiae*** | 0.075 | 0.061 | 0.073 | 0.070 | 0.110 | 0.075 | 0.049 |  | 0.066 | 0.057 | 0.032 | 0.068 |
| **9** | ***D. palpata*** | 0.082 | 0.074 | 0.073 | 0.073 | 0.117 | 0.075 | 0.064 | 0.063 |  | 0.070 | 0.067 | 0.071 |
| **10** | ***D. capreensis*** | 0.084 | 0.065 | 0.079 | 0.081 | 0.113 | 0.079 | 0.057 | 0.054 | 0.067 |  | 0.063 | 0.078 |
| **11** | ***D. geniculata*** | 0.074 | 0.065 | 0.072 | 0.070 | 0.112 | 0.075 | 0.052 | 0.031 | 0.064 | 0.060 |  | 0.066 |
| **12** | ***D. ligustica*** | 0.047 | 0.079 | 0.060 | 0.054 | 0.111 | 0.075 | 0.067 | 0.065 | 0.067 | 0.074 | 0.063 |  |
