# Supplementary material for: Molecular evolution of the pDo500 satellite DNA family in Dolichopoda cave crickets (Rhaphidophoridae)
Source: BMC Evol Biol. 2009 Dec 28;9:301. doi: 10.1186/1471-2148-9-301 (PMC2808323; doi:10.1186/1471-2148-9-301)
Supplement: Additional file 2 — Interspecific genetic distances based on pDo500 satDNA consensus sequences. Interspecific Kimura (1980) two-parameter distances (above diagonal) and uncorrected p-distance (below diagonal) of 32 derived population specific pDo500 satDNA consensus sequences from Dolichopoda [file 1471-2148-9-301-S2.DOC]

**Additional file 2.** Interspecific genetic distances based on consensus sequences. Interspecific Kimura (1980) two-parameter distances (above diagonal) and uncorrected p-distance (below diagonal) of 32 derived population specific *pDo500* satDNA consensus sequences from *Dolichopoda.*

|  | **Species** | ***1*** | ***2*** | ***3*** | ***4*** | ***5*** | ***6*** | ***7*** | ***8*** | ***9*** | ***10*** | ***11*** | ***12*** |
| --- | --- | --- | --- | --- | --- | --- | --- | --- | --- | --- | --- | --- | --- |
| **1** | ***D. schiavazzii*** |  | 0.058 | 0.047 | 0.048 | 0.146 | 0.068 | 0.065 | 0.066 | 0.058 | 0.060 | 0.060 | 0.027 |
| **2** | ***D. aegilion*** | 0.055 |  | 0.044 | 0.055 | 0.108 | 0.048 | 0.013 | 0.026 | 0.020 | 0.016 | 0.027 | 0.045 |
| **3** | ***D. linderi*** | 0.046 | 0.043 |  | 0.025 | 0.129 | 0.055 | 0.053 | 0.056 | 0.041 | 0.044 | 0.051 | 0.038 |
| **4** | ***D. bolivari*** | 0.047 | 0.053 | 0.025 |  | 0.153 | 0.060 | 0.065 | 0.059 | 0.053 | 0.056 | 0.055 | 0.037 |
| **5** | ***D. cyrnensis*** | 0.133 | 0.100 | 0.118 | 0.138 |  | 0.142 | 0.119 | 0.129 | 0.111 | 0.109 | 0.127 | 0.137 |
| **6** | ***D. bormansi*** | 0.065 | 0.046 | 0.053 | 0.057 | 0.129 |  | 0.060 | 0.057 | 0.043 | 0.051 | 0.053 | 0.056 |
| **7** | ***D. baccettii*** | 0.062 | 0.013 | 0.051 | 0.062 | 0.110 | 0.057 |  | 0.039 | 0.034 | 0.030 | 0.040 | 0.057 |
| **8** | ***D. laetitiae*** | 0.063 | 0.026 | 0.054 | 0.057 | 0.118 | 0.055 | 0.038 |  | 0.033 | 0.019 | 0.013 | 0.054 |
| **9** | ***D. palpata*** | 0.056 | 0.020 | 0.040 | 0.051 | 0.103 | 0.042 | 0.033 | 0.032 |  | 0.011 | 0.032 | 0.045 |
| **10** | ***D. capreensis*** | 0.058 | 0.016 | 0.043 | 0.054 | 0.101 | 0.049 | 0.029 | 0.019 | 0.011 |  | 0.023 | 0.047 |
| **11** | ***D. geniculata*** | 0.058 | 0.027 | 0.049 | 0.053 | 0.117 | 0.051 | 0.039 | 0.012 | 0.032 | 0.022 |  | 0.048 |
| **12** | ***D. ligustica*** | 0.027 | 0.044 | 0.037 | 0.037 | 0.125 | 0.054 | 0.055 | 0.052 | 0.044 | 0.046 | 0.047 |  |
