# Supplementary figures and images for: Molecular evolution of the pDo500 satellite DNA family in Dolichopoda cave crickets (Rhaphidophoridae)
Source: BMC Evol Biol. 2009 Dec 28;9:301. doi: 10.1186/1471-2148-9-301 (PMC2808323; doi:10.1186/1471-2148-9-301)

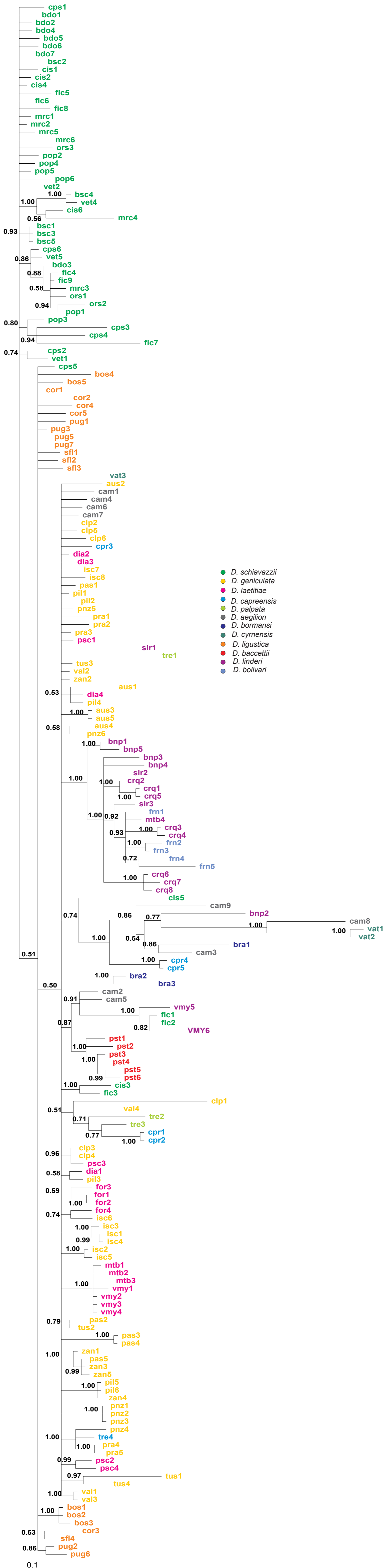

Supplement: Additional file 3 — Unrooted Bayesian phylogeny of the 199 repeats of the pDo500 satDNA family of Dolichopoda cave crickets. Posterior probabilities (PP) > 50% are given. Abbreviations for populations are as in Table 1. The Maximum Parsimony and the Maximum Likelihood analyses were congruent with the Bayesian analysis. [file 1471-2148-9-301-S3.PDF]

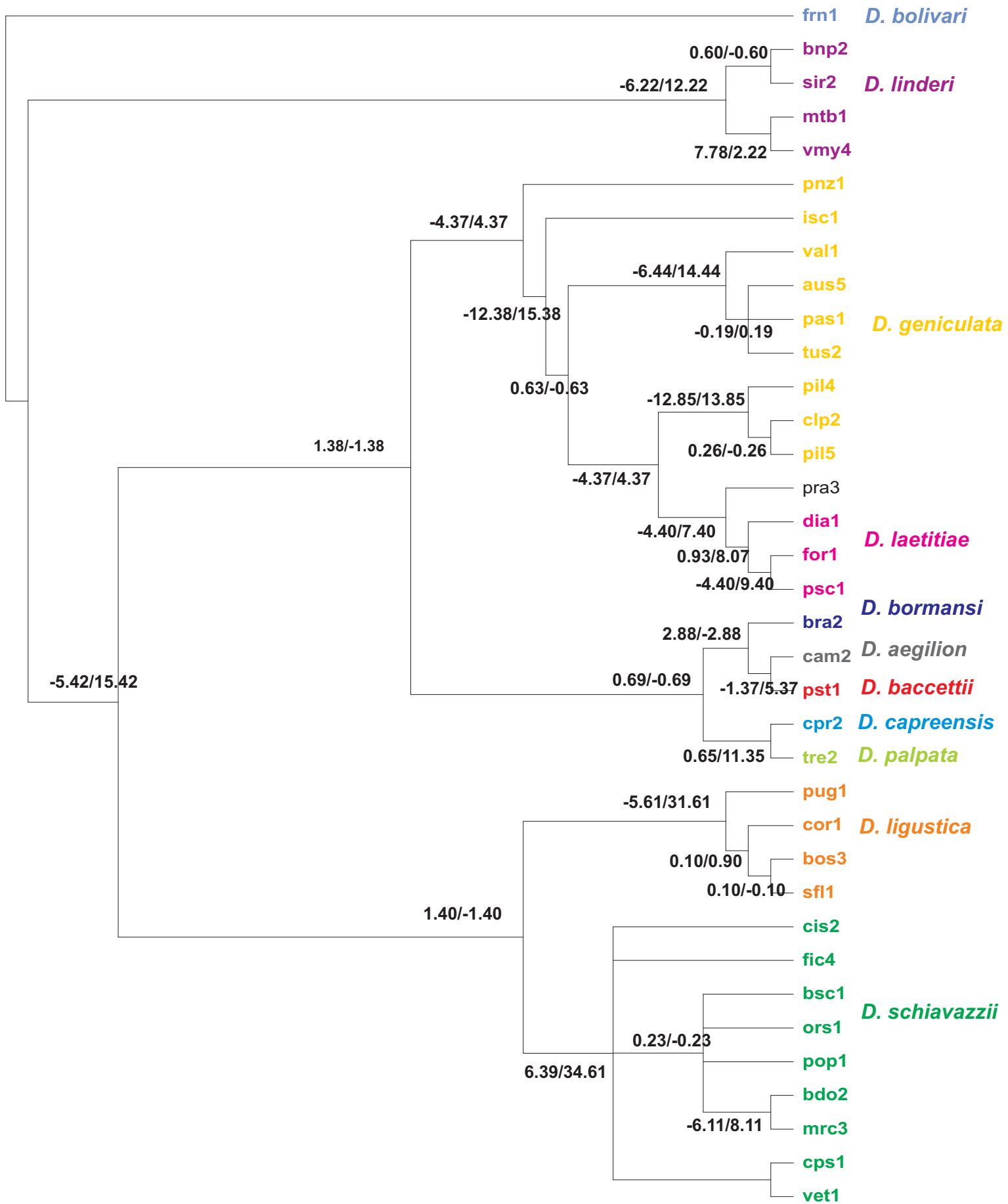

Supplement: Additional file 4 — Partition Bremer Support values for one of the most parsimonious trees from the pDo500 satDNA consensus and the mtDNA sequences. The PBS values for the two data partitions are given as satDNA/mtDNA. [file 1471-2148-9-301-S4.PDF]

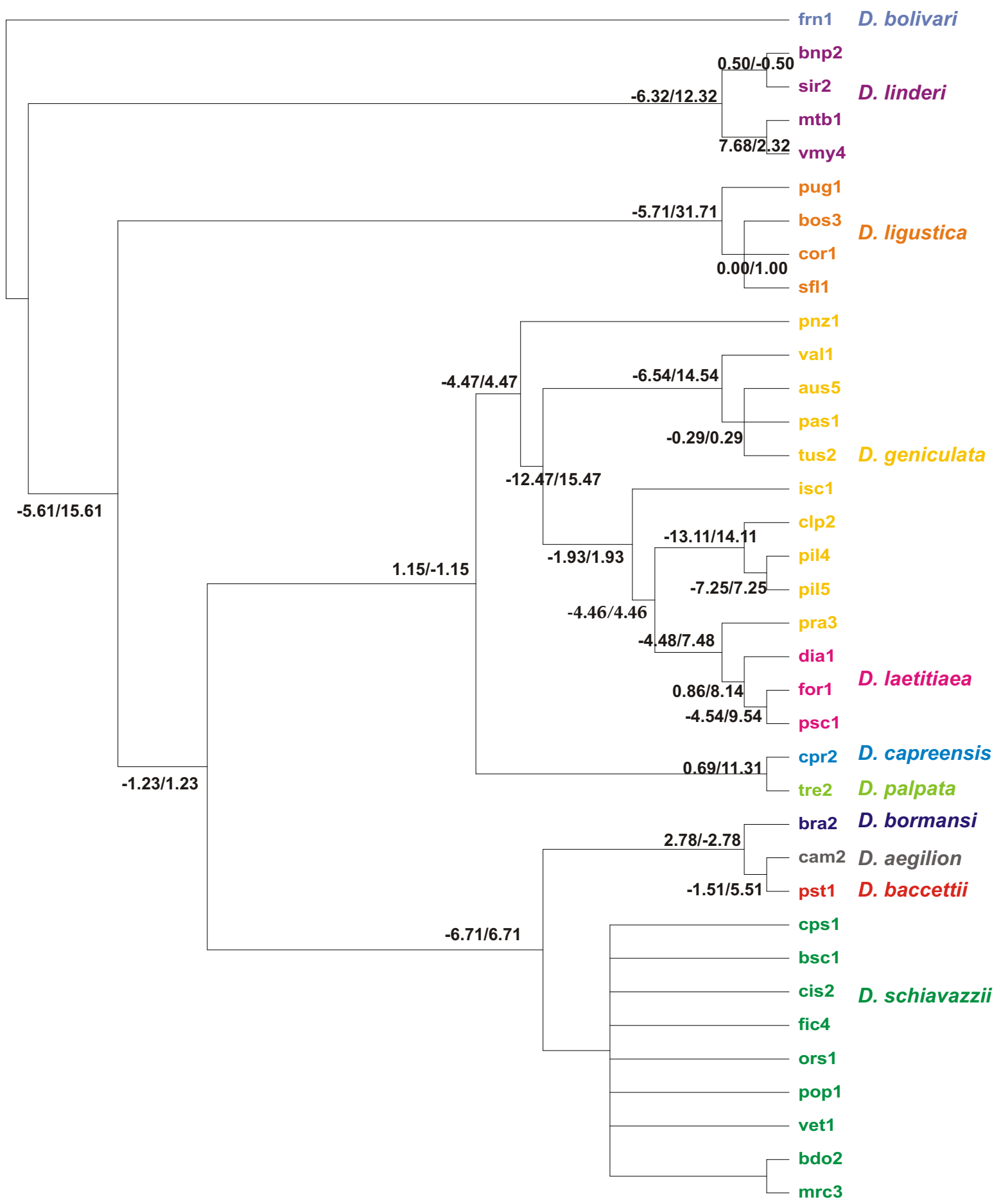

Supplement: Additional file 5 — Partition Bremer Support values for the 50% majority-rule consensus tree derived for the pDo500 satDNA consensus and the mtDNA sequences. The 50% majority-rule consensus tree is based on the MPTs from the combined parsimony analysis of the satDNA consensus and the mtDNA sequences. The PBS values for the two data partitions are given as satDNA/mtDNA. [file 1471-2148-9-301-S5.PDF]
