# Supplementary material for: Molecular evolution of the pDo500 satellite DNA family in Dolichopoda cave crickets (Rhaphidophoridae)
Source: BMC Evol Biol. 2009 Dec 28;9:301. doi: 10.1186/1471-2148-9-301 (PMC2808323; doi:10.1186/1471-2148-9-301)
Supplement: Additional file 6 — Map of the geographical distributions of Dolichopoda species included in this study. The map is showing the distribution areas for the Dolichopoda species in the West Mediterranean region. [file 1471-2148-9-301-S6.PDF]

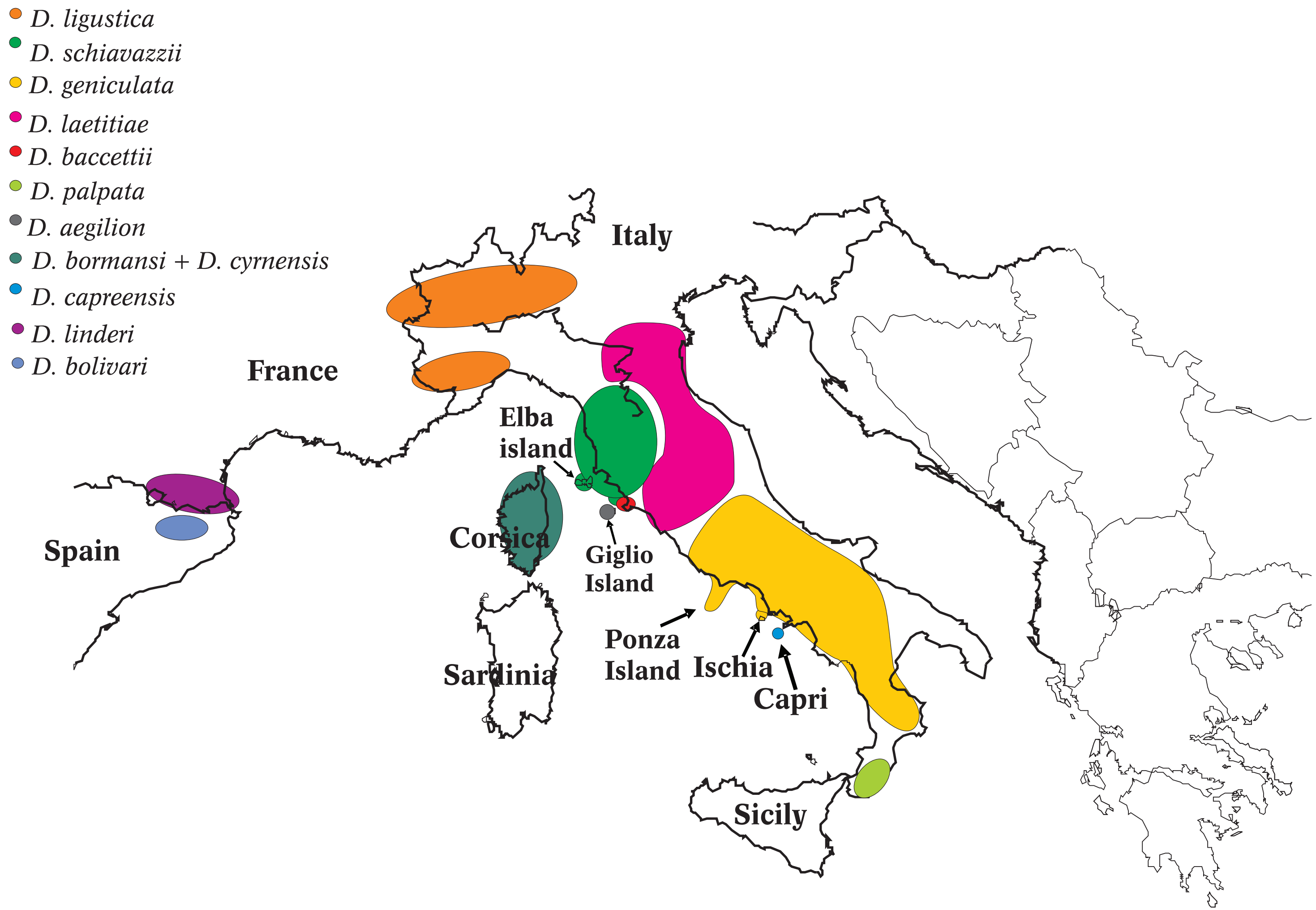
D. ligustica
D. schiavazzii
D. geniculata
D. laetitiae
D. baccettii
D. palpata
D. aegilion
D. bormansi + D. cyrnensis
D. capreensis
D. linderi
D. bolivari
Italy
France
Spain
Elba island
Corsica
Giglio Island
Sardinia
Ponza Island
Ischia
Capri
Sicily
